# Editorial: Machine Learning in Neuroimaging

**DOI:** 10.3389/fneur.2021.778765

**Published:** 2021-11-24

**Authors:** Christian Federau, Fabien Scalzo, Christopher William Lee-Messer, Greg Zaharchuk

**Affiliations:** ^1^Eigenossische Technische Hochschule (ETH) Zürich, Zurich, Switzerland; ^2^AI Medical AG, Zollikon, Switzerland; ^3^Seaver College, Pepperdine University, Malibu, CA, United States; ^4^Stanford University, Stanford, CA, United States

**Keywords:** machine learning, artificial intelligence, neural network, neuroimaging, neuroradiology

Artificial intelligence methods hold the potential of significantly and profoundly impacting neuroimaging treatment and analysis, therapeutic decision-making, and ultimately improving the patient outcome. These methods offer new computational ways to solve mathematical minimization and optimization problems, that are particularly adapted for image processing in radiology, where other methods might fail due to the complex signal characteristics and anatomical relationships. Advances in network design, processing power, availability of easy-to-use software packages, and the increasing scale of medical image databases have accelerated the developments in this exciting field. This Research Topic focuses on recent advances in the applications of machine learning and in particular deep learning to neuroimaging applications. Indeed, studies evaluating the potential applications of machine learning methods for the detection, lesion segmentation, therapeutic decision-making, and prognosis of brain diseases are still relatively sparse.

Jiang et al. for example, successfully applied convolutional neural networks to predict brain age in healthy adults from age-related neuroanatomical changes during aging process in common structural brain networks extracted from magnetic resonance images. Rebsamen et al. demonstrated, that human brain morphometry information of clinical relevance, such as subcortical volume, mean cortical thickness and curvature of cortical parcellations could be obtained within seconds using convolutional neural networks, compared to 30 min using conventional algorithms from FreeSurfer. They also observed age-related reductions in cortical thickness not only globally, but also on most of the individual parcellations. Zopes et al. demonstrated that brain segmentation of multiples anatomical substructures could be obtained within seconds using convolutional neural networks, not only on T1-weighted MRIs, but also on other contrasts such as FLAIR, diffusion-weighed MRIs, as well as on CT. They used dropout sampling for uncertainty quantification to identify corrupted input scans or low-quality segmentations, and showed that the segmentation uncertainty was slightly higher for FLAIR, diffusion-weighed MRI, and CT compared to T1-weighted MRI. Liu et al. showed in their papers that the tedious task of segmenting white matter lesions in patients with acute ischemic lesions could be well-performed by a U-Net. Alwalid et al. showed that computationally cheap, engineered radiomic features can play a role in identifying ruptured intracranial aneurysms in segmented regions of CT angiograms. Xia et al. predicted with a good performance the clinical outcome at discharge after rupture of anterior communicating artery aneurysm using a random forest. In this single center study, they used a large cohort of hundreds of patients for training, and could better predict clinical outcome from the random forest model compared to two radiologists. They also found that although aneurysm morphological parameters are significantly related to aneurysm rupture, none of the aneurysm morphological parameters they investigated was an independent risk factor of clinical outcome after rupture of anterior communicating artery aneurysm. Indeed, the aneurysm morphology is irrelevant once an aneurysm ruptures, but the extend and the localization of the bleeding, as well as on further parameters such as the patient's age, general health status, and further neurological conditions, are of importance. Luckett et al. could demonstrate that deep learning can represent, at the group level analysis, the localization of the language system from resting state functional MRIs in a cohort of 35 patients with brain tumors, compared to the localization estimated in the same patients with task-based functional MRI. Finally, Rosas-Gonzalez et al. combined in an asymmetric ensemble of U-Net-like models, 2D low-level features extraction with low memory consumption 3D contextual information extraction. They could show their asymmetric so-called ensemble of asymmetric U-Nets could to obtain similar brain tumor segmentation compared to state-of-the art U-Net while reducing the amount of data necessary for training.

The above-mentioned results published in this Research Topic confirm the great potential of machine learning methods to improve various aspects of neuroimaging (1). Applications of artificial methods to diagnostic neuroradiology are really only emerging and have a bright future (2), in particular to assist the neuroradiologists in tedious tasks such as the segmentation of multiple sclerosis (3) or stroke (4) lesions, or to help in the emergency room setting, for example in detecting hemorrhage on non-contrast CT (5).

## Author Contributions

All authors listed have made a substantial, direct, and intellectual contribution to the work and approved it for publication.

## Conflict of Interest

CF was employed by company AI Medical AG. GZ discloses funding research from GE Healthcare and the NIH, an advisory role at Biogen, and is co-founder of Subtle Medical, Inc. with an equity interest. The remaining authors declare that the research was conducted in the absence of any commercial or financial relationships that could be construed as a potential conflict of interest.

## Publisher's Note

All claims expressed in this article are solely those of the authors and do not necessarily represent those of their affiliated organizations, or those of the publisher, the editors and the reviewers. Any product that may be evaluated in this article, or claim that may be made by its manufacturer, is not guaranteed or endorsed by the publisher.
